# Anchoring wire technique for additional stent placement after endoscopic ultrasonography-guided hepaticogastrostomy

**DOI:** 10.1055/a-2643-8405

**Published:** 2025-07-25

**Authors:** Kazuki Endo, Haruo Miwa, Ritsuko Oishi, Yuichi Suzuki, Kazuya Sugimori, Kazushi Numata, Shin Maeda

**Affiliations:** 1 26437 Gastroenterological Center, Yokohama City University Medical Center, Yokohama, Japan; 2 Department of Gastroenterology, Yokohama City University, School of Medicine, Yokohama, Japan

Endoscopic ultrasonography-guided hepaticogastrostomy (EUS-HGS) has been reported to have a high technical success rate; however, severe complications occasionally occur 1 . Among these, migration of a self-expandable metal stent (SEMS) into the abdominal cavity during and after EUS-HGS is a potentially fatal complication 2 3 . In cases where the SEMS becomes partially dislodged toward the abdominal cavity, resulting in a shortened intragastric length, additional SEMS placement has been reported 4 ; however, it carries the risk of pushing the SEMS into the abdominal cavity. Herein, we report a novel anchoring wire technique during additional SEMS placement after EUS-HGS ( Video 1 ).

An additional SEMS was safely placed using the anchoring wire method for the partially dislodged SEMS after EUS-HGS.Video 1

A 71-year-old man with unresectable ampullary carcinoma was admitted for cholangitis due to occlusion of SEMS. Endoscopic retrograde cholangiopancreatography was performed; however, additional SEMS placement failed due to tumor invasion in the second portion of the duodenum. Therefore, EUS-HGS was performed after nasobiliary drainage, and a fully covered SEMS (HANARO Benefit, 8-mm, 12-cm; Boston Scientific, Marlborough, Massachusetts, USA) was deployed in B3. The following day, computed tomography images revealed that the gastric side of the SEMS had partially dislodged toward the abdominal cavity ( Fig. 1 ). An emergency endoscopic reintervention was performed for additional SEMS placement. The stent cover was penetrated using a tapered catheter, and the sufficient length of a 0.025-inch guidewire (VisiGlide 2, Olympus medical systems, Tokyo, Japan) was inserted through the mesh gap and left in the stomach as an anchoring wire. Subsequently, a second guidewire was advanced into the SEMS from the gastric end, and an additional SEMS, identical to the previous stent, was successfully deployed without stent migration ( Fig. 2 ).

**Fig. 1:**
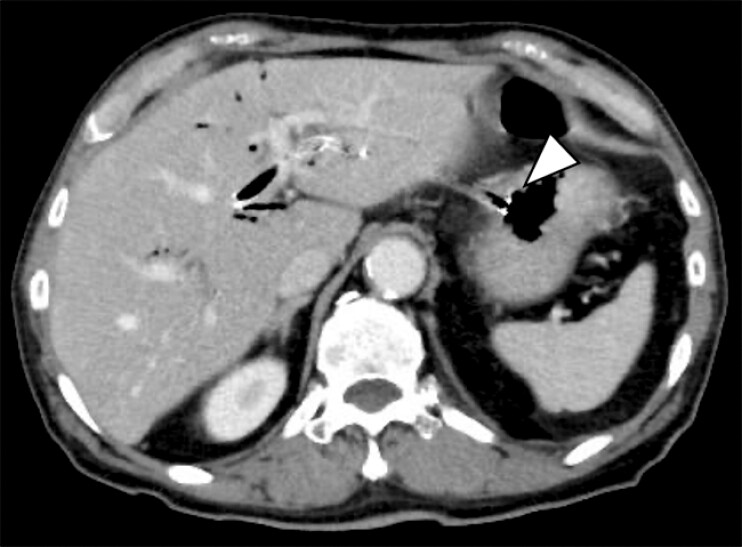
Computed tomography on the day after EUS-HGS reveals that the gastric side of the SEMS is partially dislodged toward the abdominal cavity (arrowhead).

**Fig. 2:**
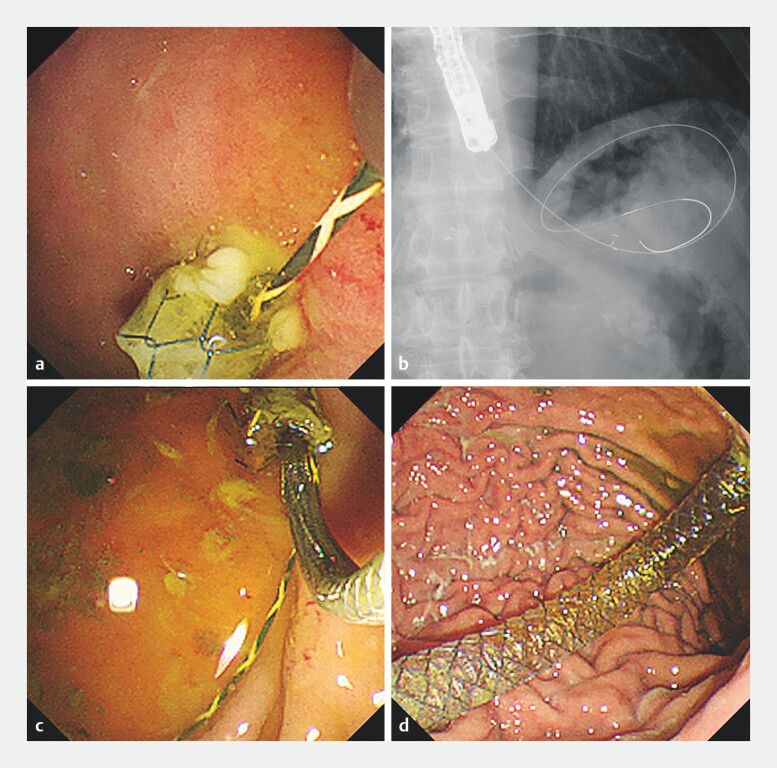
Additional SEMS placement using anchoring wire method. **a** Silicon cover of the SEMS is penetrated using a tapered catheter and a 0.025-inch guidewire is inserted through the mesh gap. **b** A sufficient length of the guidewire is left in the stomach. **c** A delivery system of the additional SEMS is inserted from the gastric end. **d** The anchoring wire is removed just before the additional SEMS placement and the additional SEMS is successfully placed.

To the best of our knowledge, this is the first report of novel anchoring wire technique to prevent SEMS migration during reintervention after EUS-HGS. This method is useful as a rescue technique for additional SEMS placement.

Endoscopy_UCTN_Code_TTT_1AS_2AH

## Notes

Endoscopy E-Videos https://eref.thieme.de/e-videos
*E-Videos* is an open access online section of the journal *Endoscopy* , reporting on interesting cases and new techniques in gastroenterological endoscopy. All papers include a high-quality video and are published with a Creative Commons CC-BY license. Endoscopy E-Videos qualify for HINARI discounts and waivers and eligibility is automatically checked during the submission process. We grant 100% waivers to articles whose corresponding authors are based in Group A countries and 50% waivers to those who are based in Group B countries as classified by Research4Life (see: https://www.research4life.org/access/eligibility/ ). This section has its own submission website at https://mc.manuscriptcentral.com/e-videos .
